# Colour volumetric display based on holographic-laser-excited graphics using drawing space separation

**DOI:** 10.1038/s41598-021-02107-3

**Published:** 2021-11-23

**Authors:** Kota Kumagai, Shun Miura, Yoshio Hayasaki

**Affiliations:** Center for Optical Research and Education (CORE), Utsunomiya University, Utsunomiya, 321-8585 Japan

**Keywords:** Applied optics, Displays

## Abstract

A volumetric display generates a graphics that can be viewed from 360$^{\circ }$ by representing the 3D information of an object as voxels in physical space. However, the natural properties of physical objects, such as 3D information and colors, and the seamless relationships between graphics and humans make it difficult to implement such displays. Here, we introduce a novel system that combines the spatial generation of femtosecond-laser-excited emission points using computer-generated holograms and beam scanning with the drawing space separation method. We demonstrate the drawing of volumetric graphics that can be color-expressed in voxel units in the air. This system enables the drawing of volumetric graphics in the air, accurate color representations, and robust graphics that are not destroyed by contact with users or objects. It also lays the foundation for the implementation of future volumetric displays.

## Introduction

Many display technologies have attempted to overcome the gap between real objects and graphics. For example, several approaches have been proposed to reproduce the 3D information of real objects. Holographic displays that use the principle of light interference enable the formation of 3D graphics that trigger depth cues in the human vision system because they contain spatial phase information. Research and development on eliminating the tradeoff between display size and viewable angle using holographic optical elements^1^ and steering-backlight units^2^, as well as developing calculation algorithms using machine learning to realize high image quality^3^, are continuing to bring dynamic holographic displays closer to a practical level. Head-mounted displays (HMDs) have seen significant development in recent years and have realized 3D image experiences that cover a user’s field of view using special wearable devices. HMDs have already been used in consumer products such as the Sony PS VR and Oculus Quest 2, which provide virtual reality applications for design, education support, and entertainment. In relation to this HMD technology, display methods with an image extended depth of field based on the focal sweep method^4^, as well as holography^5^, have been proposed for taking the next step to reducing the discomfort of viewing images as much as possible by solving vergence-accommodation conflicts. However, in these systems, because graphics are created on 2D planes, the viewable range is restricted to those plane, unlike the viewable range of an actual object. To present 3D graphics with a wide viewable range beyond those based on a flat panel display without causing user discomfort or requiring special wearable devices, methods for displaying graphics in 3D space are required.

Volumetric displays are one type of method aiming to overcome these challenges^6^. Such displays present 3D graphics by generating volume pixels (voxels) that are visualized through light emission or scattering. Because voxels are generated in space as points to form a graphics, they can leverage human depth perception and display 3D graphics that can be viewed with the naked eye. In previous studies, various systems using voxels with different optical characteristics have been reported. For light scattering, various systems utilize rotating screens^7–9^, bubbles in a high-viscosity liquid^10,11^, and water droplet arrays as voxels^12^. Such displays have the advantage of colorization of volumetric graphics because they can generate voxels with arbitrary colors by controlling the wavelength of the scattered illumination light source. Regarding methods that emit light directly from voxels, there are displays using the light emission of laser-excited rare-earth elements^13–16^ and fluorescent materials^17,18^, LEDs^19^, and aerial plasma generated by an ultrashort pulse laser^20^ as voxels. These systems are constructed with a light source or electricity source and a screen without introducing illumination light for the visualization of voxels.

Among these implementations, in recent years, display methods for drawing volumetric graphics that can express color in the air have been proposed. The colors of graphics and aerial displays without physical screens are valuable as representations of 3D information for developing display technology to fill the gap between real objects and graphics. Aerial volumetric graphics with colors have been generated by forming voxels using acoustic addresses and laser addresses. Acoustic address methods generate voxels using illuminated scattering particles that are captured by acoustic potential fields with ultrasonic-phased arrays^21,22^. Laser address methods captures a particle using photophoretic force and introduce an illumination light source to generate voxels^23^. These acoustic and laser addressing methods display aerial volumetric graphics based on the spatial scanning of particles and utilize the characteristics of scattering voxels to achieve the colorization of graphics by changing the colors of illumination lights.

We previously proposed a display system that realizes volumetric graphics in the air by combining femtosecond-laser-excited aerial voxels with a beam design technique based on computer-generated holograms (CGHs)^24^. Since the system does not keep to capture a scatted particle and enables to draw robust images that does not disappear even after contact with users and real objects, it demonstrated not only aerial volumetric image but also touch interaction with images and real-world augmented reality. These features represent powerful advantages when imagining a future in which the physical world and graphics are highly fused. Furthermore, the beam design technique based on CGHs can be applied to the parallel generation of focal points and enables the generation of multiple voxels. However, because the femtosecond-laser-excited aerial voxels emit bluish-white monochromatic light, this system does not achieve the colorization of volumetric graphics. Additionally, the size of the graphics and number of voxels that can be displayed are restricted because voxel generation in the air requires a high pulse energy. The size of the graphics depends on the scanning range of the voxel, which is determined by the focal length of the lens. In previous system, the graphics size was several millimeters because the lens having short focal length which can form high-energy density at focal point was used to generate bright voxels in the air. However, filling the drawing space with a material that can reduce the energy threshold for voxel generation will reduce the realism and interactivity of graphics because it will create a physical wall between graphics and users.

**Figure 1:**
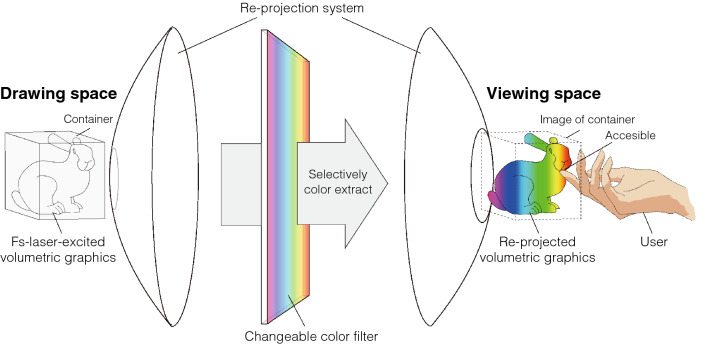
Concept of drawing space separation (DSS). The drawing space is separated from the viewing space by a re-projection system. DSS allows the arrangement of a mechanism that can change the transmitted color of light and displays a multicolor volumetric graphics by selectively extracting colors from femtosecond-laser-excited emission points. The arrangement of materials that assist in emission generation in the drawing space is possible without interfering with accessibility between the user and graphics. This figure was created using Adobe Illustrator 2021 version 25.4.1 (https://www.adobe.com/products/illustrator.html).

In this paper, we introduce a novel volumetric display that combines the spatial generation of femtosecond-laser-excited voxels using CGHs and spatial beam scanning with the drawing space separation (DSS) method. The core concept of this technique is to separate the space in which a graphics is drawn from the space that users can see by providing a re-projection method for volumetric graphics (Fig. 1). This separation allows us to implement a color extraction mechanism that can switch the transmission wavelength over time during the re-projection process to generate multicolored voxels by selectively extracting arbitrary light colors from femtosecond-laser-excited voxels with emission colors that cover the visible light range. Furthermore, this display system does not form gaps between users and graphics, even if the drawing space is filled with a material that can generate voxels with less pulse energy than air, because it generates real images that are visible to the naked eye. Aerial re-projected voxels are implemented through the combination of emission points generated by the focused irradiation of a femtosecond laser on a material and aerial projection with two parabolic mirrors facing each other. Because our drawing method using CGHs enables the formation of multiple focal points simultaneously, the number of voxels per unit time is increased. Our system makes it possible to draw volumetric graphics that can express colors in the air and establishes a new foundation for volumetric display implementation.

## Results

### Generation of multicolored aerial voxels

**Figure 2:**
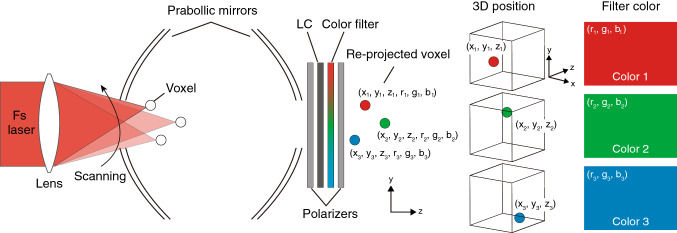
Spatial generation of aerial voxels with arbitrary colors. A voxel re-projected by parabolic mirrors is controlled in terms of its spatial position (*x*, *y*, *z*) color using beam scanning and a liquid crystal (LC) color filter (*r*, *g*, *b*). This figure was created using Adobe Illustrator 2021 version 25.4.1 (https://www.adobe.com/products/illustrator.html).

Figure 2 presents a schematic diagram of DSS used to generate voxels with different colors. In this study, the generation of multicolored aerial voxels based on DSS was implemented via re-projection using two centrally apertured parabolic mirrors and an LC color filter. The aerial re-projection method, which was reported in a US patent 50 years ago^25^, uses two parabolic mirrors that are placed with their concave sides facing toward each other such that their vertices are separated by the focal length^26^. An object placed on a bottom mirror is projected as a real image in the region of the aperture of an upper mirror. This method has also been utilized to generate 3D images using a CGH displayed on an SLM^27^, and a high-speed projected spinning diffuser^28^ in place of a physical object.

In our system, volumetric graphics are generated by a focused femtosecond laser in a drawing space generated by creating an aperture in the bottom mirror. Voxels are generated in the aperture of the parabolic mirror and are re-projected into the air through the aperture in the other parabolic mirror on the opposite side. Only arbitrary emission colors can be extracted by the LC color filter used in the re-projection process. The generation positions of the voxels are spatially arranged by scanning the deflection angle and focal length of the femtosecond laser. The proposed system realizes the drawing of multicolored volumetric graphics by changing the spatial generation position $(x_i, y_i, z_i)$ of the *i*th voxel and the color value $(r_i, g_i, b_i)$ assigned to the LC color filter with time synchronization.

### Evaluation of a voxel

**Figure 3:**
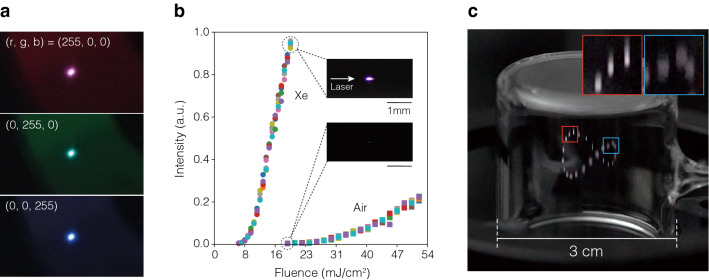
Femtosecond-laser-excited voxels. (**a**) Voxels generated by the re-projection of femtosecond-laser-excited emissions in the air. Three RGB emission colors were achieved by varying the values of the LC color filter (*r*, *g*, *b*) to R(255, 0, 0), G(0, 255, 0), and B(0, 0, 255). (**b**) Emission brightness generated in Xe and ambient air versus the irradiated fluence intensity. Macro images of each emission point were captured at 19 mJ/${\text {cm}}^{2}$. (**c**) Example graphics in a Xe-filled glass cell. Some of the emission points at different positions are magnified.

Figure 3a shows the voxels in the viewing space generated by a volumetric display based on DSS. These voxels are aerial re-projections of the emission points obtained when a femtosecond laser is irradiated with a lens with a focal length of 50 mm at a light intensity of 52 mJ/${\text {cm}}^{2}$ in an ambient-air-filled drawing space. These images were captured by a camera ($\alpha$7III, Sony) with an exposure time of 20 ms. By changing the pixel values (*r*, *g*, *b*) assigned to the LC color filter, the voxels can effectively extract the emission colors corresponding to the filter. Therefore, we have confirmed the effectiveness of DSS for the generation of aerial voxels with arbitrary colors. The viewable angle range of the voxels is 360$^{\circ }$ in the horizontal direction and a specific range in the vertical direction, which is described in the Supplementary Note 1 and Fig. 1.

We focused on xenon gas as a material for filling the drawing space based on its high emission brightness and broadband wavelength covering the visible region, as well as the reduction of the energy threshold required for excitation^29,30^. Figure 3b presents the brightness of the emission point generated by the focused femtosecond laser with respect to the incident fluence. Here, Xe is enclosed in the glass cell as an excitation target. The brightness is calculated from the total value of $30 \times 30$ pixels, including the emission point, based on images captured by a charge coupled device (CCD) camera (DFKZ12G445, The Imaging Source) and was observed five times for each irradiation intensity. The emission of the Xe exhibits a significant change in brightness with respect to the irradiation intensity. Compared to the luminescence of air, it is approximately 200 times brighter at 19 mJ/${\text {cm}}^{2}$, where the emission of air begins, and the threshold intensity for generating emissions is reduced by up to 34%. An example of an graphics in Xe drawn via the spatial scanning of the emission point is presented in Fig. 3c. We confirmed that it is possible to draw bright voxels that can be observed under ambient room lighting. Therefore, Xe was adopted as the drawing space gas for the remainder of our evaluations.

### Experimental setup

**Figure 4:**
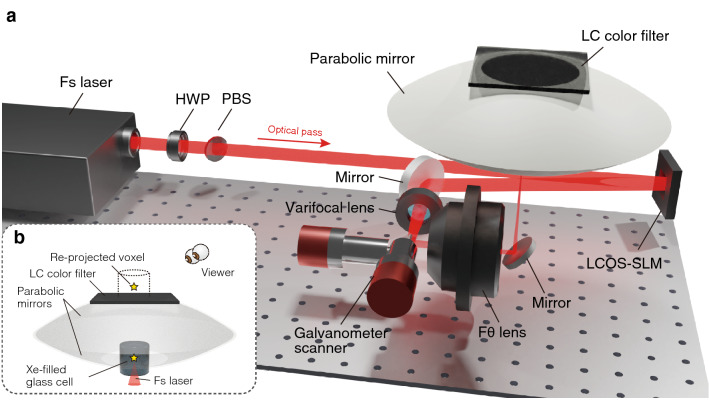
Design of a volumetric display system based on DSS. (**a**) A femtosecond-laser-excited emission point is spatially scanned in an aperture on the bottom parabolic mirror by a beam scanning system and enhanced by a CGH on an LCOS-SLM. A half-wave plate (HWP) and a polarization beamsplitter (PBS) are used to control pulse energy of the femtosecond laser. The photograph of the actual system is shown in the Supplementary Fig. 2. (**b**) The emission point is generated as a voxel in the viewing space by the re-projection system, and its emission brightness is enhanced by the introduction of a glass cell filled with Xe gas. This figure was created using Blender version 2.91 (https://www.blender.org/).

Figure 4 presents our experimental setup for testing the proposed system. In our experiments, volumetric graphics were rendered based on the persistence of vision occurring during the high-speed spatial scanning of aerial emission points excited by a focused femtosecond laser. The phase of the femtosecond laser pulses were controlled by a liquid-crystal-on-silicon spatial light modulator (LCOS-SLM) on which a CGH was displayed. We generated voxels on the drawing space after it passed through a 3D beam scanning system. The generated voxels in the drawing space were selectively extracted from original voxel emissions with arbitrary colors using a re-projection system constructed from two parabolic mirrors and an LC color filter. The phase control of the laser by the CGH contributes to the parallel generation of focal points and increases the number of voxels that can be formed per unit time. The 3D beam scanning system performs voxel arrangement to generate volumetric graphics by enabling spatial scanning of a focal point.

The femtosecond laser (Micra and Legend Elite Duo, Coherent) has a center wavelength of 800 nm and repetition frequency of 1 kHz with an average power of 7 W. The LCOS-SLM (X13139-02, Hamamatsu Photonics K.K.) supports $1272 \times 1024$ pixels with a pixel pitch of 12.5 ${\upmu }$m and is driven by an 8 bit signal with a refresh rate of about 10 Hz. The polarization direction of the laser incident on the LCOS-SLM was aligned with the orientation of the LC to obtain the maximum diffraction efficiency. The 3D beam scanner was composed of a galvanometer scanner (GM-1010, Canon) and a varifocal lens (EL-16-40-TC-VIS-5D-C, Optotune). The galvanometer scanner has an available beam deflection angle of 40$^{\circ }$ and enables the scanning of focal points in the horizontal direction. The varifocal lens has an aperture of 16 mm and variable focal length region of $-500$ to $+333$ mm, which allowed us to control the axial position of the focal point. The LC color filter was placed immediately after the aperture of the parabolic mirror on the re-projected graphics side and can switch the filter color. A Xe-filled cylindrical glass cell with a diameter and height of 30 mm was employed as a drawing space.

### Drawing volumetric graphics

**Figure 5:**
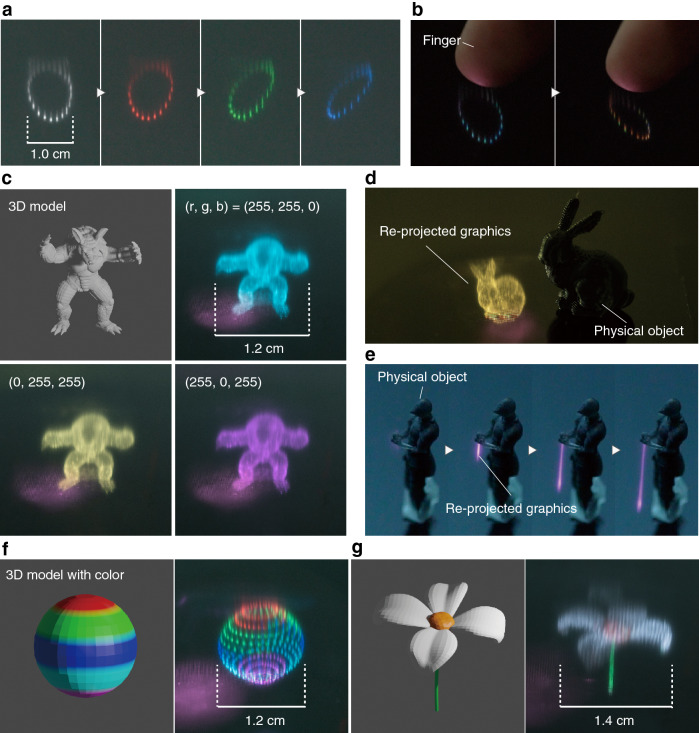
Graphics in a volumetric display based on DSS. A sequence of rotating Lissajous figures with (**a**) three different colors and (**b**) touched by a finger. (**c**) Armadillo and (**d**) Stanford bunny generated beside a physical bunny. These models were provided by the Stanford 3D Scanning Repository (http://graphics.stanford.edu/data/3Dscanrep/). (**e**) Physical knight with a light saber. The 3D model of the knight was taken from Knight Statues (https://www.thingiverse.com/thing:4198705) under the Creative Commons license CC BY 4.0 (https://creativecommons.org/licenses/by/4.0/). Images of a (**f**) sphere and (**g**) flower with multicolored voxels. The flower was taken from https://free3d.com/3d-model/flower-9941.html.

Aerial volumetric graphics drawn by the proposed display system are shown in Fig. 5. For all graphics, the femtosecond laser was set to a repetition frequency of 1 kHz and pulse duration of 34 fs immediately after the laser was emitted. The graphics in Fig. 5a was drawn while switching the LC color filter to different single colors and only the emission color selected by the filter was observed (see Supplementary Movie 1). The light observed above the rendered graphics was the reflected light from the surface of the Xe-filled glass cell. One can see that the proposed system is capable of displaying a centimeter-order image with an arbitrary color, which is visibly bright under ambient room lighting. Additionally, as shown in Fig. 5b, DSS converts the glass cell filled with Xe into a real image in the viewing space, which enables display without a gap between the user and image. These graphics were drawn using an irradiated pulse energy of 271 ${\upmu }$J and observed by a camera ($\alpha$7III, Sony) at 20 fps.

Figure 5c,d show an graphics rendered by pulse irradiation at 203 ${\upmu }$J using the vertex coordinates of a 3D model. This graphics was captured with an exposure time of 5 s. Volumetric graphics were generated by stacking 2D cross sections of 3D point cloud data sliced into layers. The scanning paths were set to the shortest scanning distances between the vertices in each layer (see “Methods”). Figure 5c presents a volumetric graphics consisting of 5734 voxels, which were rendered using color filters with different single colors of cyan, magenta, and yellow. The graphics consisting of 2603 voxels presented in Fig. 5d was drawn next to a 3D-printed physical object. These results demonstrate that the proposed system can display a volumetric graphics with a detailed shape in the air with the desired color while establishing a seamless relationship between a physical object and graphics. Additionally, we demonstrated real-world augmented reality by forming a 3D saber, as shown in Fig. 5e (see Supplementary Movie 2). This saber of light was drawn with a pulse energy of 219 ${\upmu }$J, and observed in real time at 20 fps. Figure 5f,g present images rendered based on 3D point cloud data with different color values for each vertex obtained by the scanning path calculation method. Each image was captured with an exposure time of 25 s. The sphere in Fig. 5f is composed of 472 voxels and has seven different color values. The flower in Fig. 5g, which is composed of 1206 voxels, has different colors for the stem, petals, and center. The irradiation pulse energies used for rendering Fig. 5f,g were 203 ${\upmu }$J and 117 ${\upmu }$J, respectively. Overall, we have demonstrated a volumetric display based on DSS that enables coloring on a voxel-by-voxel basis by temporally synchronizing the voxel generation positions with the color switching of the LC filter.

### Drawing graphics with the CGH

**Figure 6:**
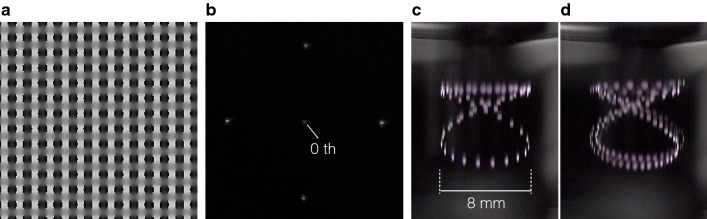
Parallel voxel generation via the CGH. (**a**) CGH calculated for a target pattern of four focal points designed to have a maximum spatial frequency $\nu = 4$ lp/mm. (**b**) Focal points generated in parallel by a lens with a focal length of 200 mm captured by a CCD image sensor. Volumetric Lissajous figures drawn with (**c**) a single focal point and (**d**) four focal points generated in parallel. These images were generated in a Xe-filled glass cell and captured in the form of movies at 20 fps.

The contribution to increasing the number of voxels per unit time for the parallel generation of focal points using a CGH is shown in Fig. 6. Figure 6a presents the CGH applied to the drawn graphics. By displaying it on the LCOS-SLM, grayscale values of 0–255 can be assigned to the light values with a phase modulation of 0–2$\pi$. This CGH was optimized through 50 Fourier iterative calculations based on the Gerchberg–Saxton method^31^ and it controls the light to form four focal points in conjunction with the lens, as shown in Fig. 6b. Each focal point is arranged to be separated by 284 ${\upmu }$m from the optical axis in a drawing space with a varifocal lens at an infinite distance. Figure 6c,d present volumetric Lissajous figures drawn with a single focal point and four parallel focal points generated in parallel by the CGH, respectively. These graphics were generated in a Xe-filled glass cell and captured in form of movies at 20 fps. These results indicate that the parallel focal points generate a graphics in which voxels are filled at a higher density compared to the single focal point for a graphics generated in one frame. We have demonstrated that the parallel focal points generated by the CGH contribute to an increase in the number of voxels generated per frame and that they can be applied to drawing a volumetric graphics.

## Discussion

This paper proposed a volumetric display system based on DSS and described concepts, principles of color representation, system implementations, and demonstrations of graphics drawing. Our proposed display enables emission color extraction from femtosecond-laser-excited voxels and the filling of a medium that contributes to the improvement of the generation efficiency of voxels in terms of incident pulse energy. It also generates volumetric graphics that can represent colors. Furthermore, the drawn images have no boundaries in the real space and are not destroyed if an object or user comes into contact with them, which is a useful feature for interactions with users and objects.

The emission colors of voxels can be extracted as desired using a re-projection system consisting of a pair of parabolic mirrors and an LC color filter. The switching speed of the emission colors is determined by the refresh rate of the filter. In the proposed system, a filter with a refresh rate of 60 Hz was adopted and it was possible to extract different colors every 17 pulses using a light source with a repetition frequency of 1 kHz. The application of a color extraction device with a switching speed comparable to that of laser repetition, which is not limited to an LC, and the development of scanning methods that generate voxels for each color will facilitate further expansion of the color expression of the proposed system. In this study, we used the temporal color switching of the filter. However, it is noteworthy that a spatially color-coded filter can facilitate the generation of graphics with different colors depending on the viewing angle.

The use of Xe as a drawing space significantly contributed to the reduction of the pulse energy required for voxel generation and the improvement of brightness, resulting in an increase in graphics size. The graphics size is determined by the scanning range of the voxels and the size of the drawing space. In the proposed system, the size is restricted by the cylindrical glass cell filled with Xe (3.0 cm in diameter $\times 3.0$ cm in height) and the visible range of the parabolic mirror, rather than by the scanning range. The scanning range $S = f\theta$ in the horizontal direction, which is determined by the combined focal length *f* = (57–117) mm of the variable lens and the deflection angle $\theta = \pm 20^{\circ }$ of the galvanometer scanner, is 40–81 mm. Therefore, the graphics can be drawn in a range of at least 40 mm$^{2}$ in the horizontal direction and 59 mm in the axial direction. Considering the effectiveness of Xe for improving voxel generation efficiency, we anticipate that it will be possible to increase the scanning range using a lens with a greater focal length. Additionally, the viewable angle range of graphics is $360^{\circ }$ in the horizontal field of view around the optical axis and approximately $10^{\circ }$ in the vertical field of view. Real-time drawing requires even faster scanning in the horizontal direction, particularly when using the layer-by-layer method. In future work, methods such as the split drawing of volumetric graphics with multiple beam access are expected to be an effective approach to achieving real-time drawing.

The parallel generation of focal points by a CGH improved the number of voxels per unit time in the generated graphics. The number of parallelized voxels *P* is determined by $P = E_{max} TD/E_{th}$, where $E_{max}$ is the maximum pulse energy of the light source, *T* is the transmittance of the entire system, *D* is the diffraction efficiency of the CGH, and $E_{th}$ is the pulse energy required for the voxel generation threshold. In the proposed system, $E_{max}$ = 7 mJ, *T* = 81%, and $E_{th}$ = 0.004 mJ, and when a focusing pattern with *D* = 50% was used, we have *P* = 708 voxels. Therefore, for real-time drawing with a refresh rate of $f_r$ = 20 Hz, the number of voxels *N* that can be generated per frame is 35,400 voxels as estimated by $N = Pn/f_r$, where *n* is the number of voxels that can be generated in 1 s, which depends on the repetition frequency of the light source. *n* can be further improved by using a femtosecond laser with a higher repetition frequency. The demonstration in Fig. 6 showed a result of *P* = 4 which is less than the number of estimated *P* because the irradiated pulse energy was adjusted within the range that does not damage the varifocal lens. In order to bring *P* closer to the estimated value, it is effective to use a varifocal lens with higher light resistance. In addition, it contributes to improvement of *P* to combine the proposed method with a dynamic optimization method for the diffraction efficiency of CGHs^32^.

Volumetric 3D information, color representations, seamless relationships with real space, and robustness that are not affected by contact with users or objects are important and powerful elements in the development of displays that fill the gap between graphics and real objects. As one configuration of a volumetric display that addresses these elements simultaneously, we believe that the proposed system based on DSS is effective and is a technology that can accelerate the development of the field.

## Methods

### Drawing of multicolor volumetric graphics

**Figure 7:**
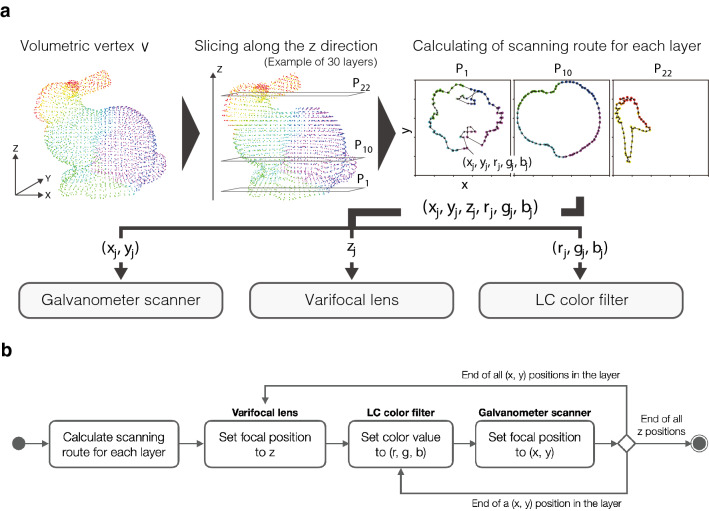
Procedure for voxel generation route acquisition. (**a**) Volumetric vertex data are sliced in layers along the z direction. The optimal scanning route is applied to the galvanometer scanner (*x*, *y*), varifocal lens *z*, and LC color filter (*r*, *g*, *b*). The 2D cross sections ${\text {P}}_{1}$, ${\text {P}}_{10}$ and ${\text {P}}_{22}$ of a 30-sliced Stanford bunny with different vertex colors are presented as an example. (**b**) Synchronous operation flow to apply the 3D coordinates and color patterns to each device. This figure was created using Adobe Illustrator 2021 version 25.4.1 (https://www.adobe.com/products/illustrator.html).

The rendering of volumetric images with arbitrary colors per voxel is performed by generating application orders of 3D coordinates and color patterns for the beam scanner and LC color filter, respectively, as shown in Fig. 7a. A 3D point cloud consisting of *M* voxels is denoted as $\varvec{V}=\{v_1, v_2,\ldots , v_M\}$, where $v_i$ is a vertex with a spatial position and RGB values $(x_i, y_i, z_i, r_i, g_i, b_i)$. First, the *z* value of $\varvec{V}$ is quantized to the desired number of layers, where *z* is the coordinate axis that matches the optical axis direction of the display system (Fig. 7a shows an example of a point cloud quantized to 30 layers along the z axis).

In the sliced 3D point cloud with a quantization of *z*, a layer includes vertexes ${\varvec{P}} = \{p_1, p_2, \ldots , p_N\}$, where $p_j$ has a 2D position and RGB values $(x_j, y_j, r_j, g_j, b_j)$. Next, the optimal scanning order of P in the layer is calculated based on the distance $d(p_j, p_k)$ between the selected vertices $p_j$ and $p_k$, which is calculated from their 2D coordinates. The calculation of the optimal scanning route is performed by minimizing the length of the route as follows:

1 $$\begin{aligned} \sum ^{N-1}_{j=1} d(p_j, p_{j+1})+d(p_N, p_1), \end{aligned}$$

where the Christfides algorithm^33^ is applied to all layers. The display system is given the shortest scanning order obtained for each *z* according to the operation flow as shown in Fig. 7b, yielding (*x*, *y*) values for the galvanometer scanner, *z* values for the varifocal lens, and (*r*, *g*, *b*) values for the LC color filter. In this manner, the system can draw volumetric graphics with multicolored voxels.

## Supplementary Information

Supplementary Information.

Supplementary Movie 1.

Supplementary Movie 2.
