# Supplementary material for: Colour volumetric display based on holographic-laser-excited graphics using drawing space separation
Source: Sci Rep. 2021 Nov 23;11:22728. doi: 10.1038/s41598-021-02107-3 (PMC8611029; doi:10.1038/s41598-021-02107-3)
Supplement: Supplementary file 1 — Supplementary Information. [file 41598_2021_2107_MOESM1_ESM.pdf]

Supplementary Information

Kota Kumagai, Shun Miura, Yoshio Hayasaki

Center for Optical Research and Education (CORE), Utsunomiya University

Supplementary Note

Supplementary Note 1: The angular light distribution of a re-projected voxel in the viewing space was estimated by the peak pixel value of the image taken by the CCD camera (DFKZ12G445, The imaging source) and shown in Fig. 2. As shown in Fig. 2(a), the CCD camera which was placed on the semicircular slider stage observed the voxels in 10° increments towards the optical axis direction of the laser. The plane including the aperture of the parabolic mirror was set to 0°. A voxel was re-projection of an emission point generated by focused femtosecond laser in an ambient-air-filled drawing space using the LC color filter $(r, g, b) = (255, 255, 255)$. Figure 2(b) shows the brightness of a voxel observed from different angle. The brightness were measured for voxels generated at different depths of 0 mm, 6 mm, and 12 mm in the axial direction in the drawing space. The depth was set to 0 mm at the position corresponding to the parabolic aperture on the laser incident side, and the propagation direction of the laser was set to positive. A voxel in the viewing space with a horizontal field of view of 360° are observed with a specific field of view in the vertical field of view, and the field of view shifts as the height of the generation position changes. Therefore, the user can view a graphics with a height of 12 mm near the center of the aperture within a range of about 10°.

Supplementary Figures

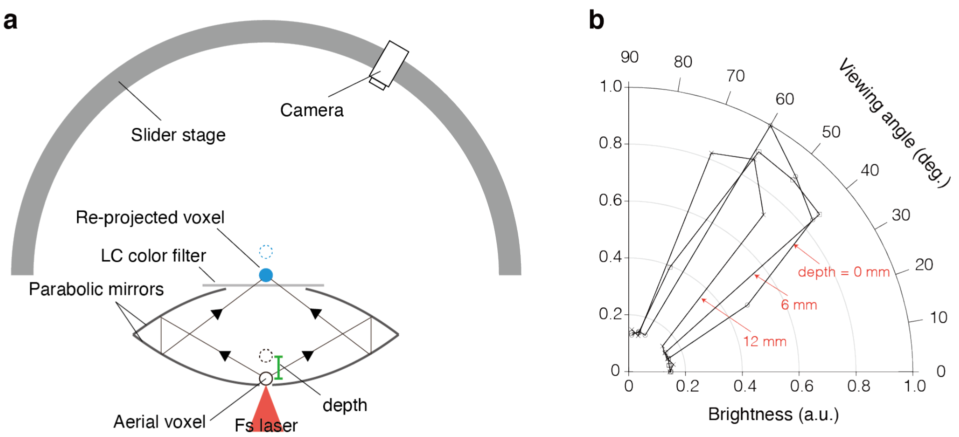

Supplementary Figure 1: Angular light distribution of a voxel. (a) Experimental setup for observing a re-projected voxel with different angle. **(b)** Brightness of a re-projected voxel versus the different viewing angle.

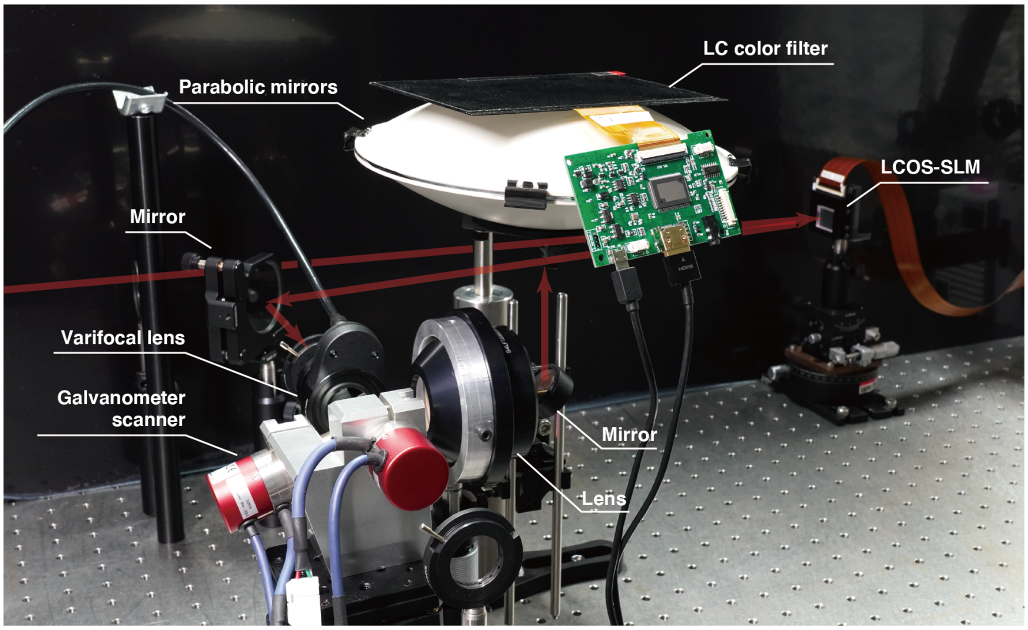

Supplementary Figure 2: Photograph of a volumetric display system.

Supplementary Movies

Supplementary Movie 1: Aerial graphics of rotating ring. The graphics that rotated while changing its color was rendered beside a 3D printed physical bunny. The bunny object was created using the 3D model in the Stanford 3D Scanning Repository (`http://graphics.stanford.edu/data/3Dscanrep/`).

Supplementary Movie 2: Knight statue with a light saber. The volumetric display augmented the knight statue with a light saber which was represented by aerial graphics. The knight was printed using Knight Statues created by mz4250 (`https://www.thingiverse.com/thing:4198705`) under the Creative Commons license CC BY 4.0 (`https://creativecommons.org/licenses/by/4.0/`).
